# Pseudohypoparathyroidism Type 1A-Subclinical Hypothyroidism and Rapid Weight Gain as Early Clinical Signs: A Clinical Review of 10 Cases

**DOI:** 10.4274/jcrpe.2743

**Published:** 2016-12-01

**Authors:** Simon Kayemba-Kay’s, Cedric Tripon, Anne Heron, Peter Hindmarsh

**Affiliations:** 1 Victor Jousselin Hospital, Clinic of Pediatrics and Neonatal Medicine, Pediatric Endocrinology Unit, Dreux, France; 2 Poitiers University Hospital, Clinic of Pediatrics, Poitiers, France; 3 Victor Jousselin Hospital, Clinical Research Unit, Dreux, France; 4 University College London and Institute of Child Health, Developmental Endocrinology Research Group, London, United Kingdom

**Keywords:** Pseudohypoparathyroidism, Subclinical hypothyroidism, early obesity, early signs

## Abstract

**Objective:**

To evaluate the clinical signs and symptoms that would help clinicians to consider pseudohypoparathyroidism (PHP) type 1A as a diagnosis in a child.

**Methods:**

A retrospective review of the medical records of children diagnosed by erythrocyte Gsα activity and/or GNAS1 gene study and followed-up for PHP type 1A. Clinical and biochemical parameters along with epidemiological data were extracted and analyzed. Weight gain during infancy and early childhood was calculated as change in weight standard deviation score (SDS), using the French growth reference values. An upward gain in weight ≥0.67 SDS during these periods was considered indicative of overweight and/or obesity.

**Results:**

Ten cases of PHP type 1A were identified (mean age 41.1 months, range from 4 to 156 months). In children aged ≤2 years, the commonest clinical features were round lunar face, obesity (70%), and subcutaneous ossifications (60%). In older children, brachydactyly was present in 60% of cases. Seizures occurred in older children (3 cases). Short stature was common at all ages. Subclinical hypothyroidism was present in 70%, increased parathormone (PTH) in 83%, and hyperphosphatemia in 50%. Only one case presented with hypocalcemia. Erythrocyte Gsα activity tested in seven children was reduced; GNAS1 gene testing was performed in 9 children. Maternal transmission was the most common (six patients). In three other cases, the mutations were de novo, c.585delGACT in exon 8 (case 2) and c.344C>TP115L in exon 5 (cases 6&7).

**Conclusion:**

Based on our results, PHP type 1A should be considered in toddlers presenting with round face, rapid weight gain, subcutaneous ossifications, and subclinical hypothyroidism. In older children, moderate mental retardation, brachydactyly, afebrile seizures, short stature, and thyroid-stimulating hormone resistance are the most suggestive features.

**WHAT IS ALREADY KNOWN ON THIS TOPIC?**

Pseudohypoparathyroidism type 1A is a rare heterogeneous disorder characterized by multiple end-organ resistance to hormones that share Gs protein-coupled receptors as signaling pathway. Age at its diagnosis is extremely variable.

**WHAT THIS STUDY ADDS?**

This study analyzes the clinical and biochemical presenting features to identify those that should raise clinicians’ suspicion and lead to the diagnosis work-up.

## INTRODUCTION

Pseudohypoaparathyroidism (PHP) type 1A is a rare genetic disorder with autosomal dominant transmission and parental imprinting, characterized by the target-organ unresponsiveness to hormones that share Gs protein-coupled receptors as the signaling pathway. Globally, PHP constitutes a heterogeneous group of disorders that have in common resistance to the action of parathyroid hormone (PTH) (1,2,3). Its prevalence has been estimated, in Japan, around 3.4 cases per million population (4).

The entity is classified into two main types: type 1 and type 2. Type 1 PHP is, in turn, divided into three subtypes 1A, 1B, and 1C, each with different pathogenesis, phenotype, and genetic findings. Overall, the three PHP type 1 subtypes result from the heterozygous loss-of-function mutation in exon 1-13 of the gene encoding Gsα or from imprinting abnormalities in the stimulating G protein, the α-subunit (Gsα) of which constitutes the signaling protein for several hormones [PTH, thyroid-stimulating hormone (TSH), gonadotropins, and glucagon] and neurotransmitters actions (2,3).

Typically, patients with PHP type 1A demonstrate laboratory findings of resistance to PTH, TSH, gonadotropins, and growth hormone-releasing hormone (GHRH), and decreased Gsα activity (≤50%) (1,2,5).

PHP type 1A patients present with typical features termed albright hereditary osteodystrophy (AHO), a constellation of short stature, round face, brachydactyly, brachymetacarpy, centripetal obesity, subcutaneous ossifications, and variable degree of mental or developmental delay (1,2,3).

From the molecular standpoint, AHO results from a heterozygous mutation of the GNAS gene encoding the G-stimulatory subunit (Gsα) located at chromosome 20q13.2. This mutation leads to a loss of expression or function of Gsα which impairs the transmission of stimulatory signals to adenylate cyclase with limitation of cyclic AMP generation necessary for hormone action (6). Consequent to the GNAS gene being subject to imprinting, AHO patients show variable hormone unresponsiveness: those with mutations on maternally inherited alleles manifest multiple hormone resistance (PTH, TSH, gonadotropins, GHRH, and glucagon) leading to PHP type 1A, while patients with mutations on paternally inherited alleles have phenotypic features of AHO without hormonal resistance (pseudopseudoparathyroidism) in consequence to Gsα expression from the paternal allele being normally suppressed because of imprinting in hormone-target tissues (6,7).

PHP type 1A is often diagnosed late due to the high variability in the age at which its characteristic features become clinically apparent (8,9).

In early infancy, features may be subtle and extremely variable ranging from a classical round face, subcutaneous ossifications, seizures, subclinical hypothyroidism to mild delay in acquisition of milestones (8,10,11,12,13), while other manifestations such as brachymetacarpia, short stature, and mental retardation tend to become apparent relatively late in childhood. Diagnosing PHP type 1A in early life relies, therefore, on strong clinical expertise and a high index of suspicion.

We reviewed a regional case series of 10 children diagnosed with PHP type 1A to identify the early signs and symptoms that might suggest a diagnosis of PHP in children.

## METHODS

We conducted a retrospective regional search of all patients diagnosed with PHP type 1A in all Pediatrics Departments of the Poitou-Charentes region in France, via each Hospital’s medical informatics department. From each medical record of identified cases, we considered only those with a confirmed diagnosis either by erythrocyte Gsα activity studies performed as previously described (5,14,15), or by molecular biology studies of GNAS1 gene (performed by commercial laboratories), or both. We extracted from each identified medical record, data on date and place of birth, birth weight (BW), birth length (BL), family history of short stature and/or relevant medical history, patient’s phenotypic features, age at which the first significant symptoms and signs became apparent, and the age at which the diagnosis was made.

Each patient’s gain in body weight during infancy (0 to 2 years) and early childhood (3 to 6 years) was calculated as changes in weight standard deviation score (SDS) using the French growth reference data (16). Body mass index (BMI) was calculated by dividing body weight in kilogram by height in meter squared and then compared against the same French reference (expressed as 5^th^, 50^th^, 85^th^ and 95^th^ centiles). Upward gain in weight ≥0.67 SDS during infancy or early childhood was considered indicative of overweight, as previously reported (17). Similarly, an increase in BMI at/or above 85th centile during the same periods was considered as indicative of overweight, and a BMI at/or above 95th as indicative of obesity.

We also extracted from each medical record the results of biochemical parameters (thyroid function tests, Gsα activity test, molecular gene study results, growth hormone (GH) test results when performed, luteinizing hormone, follicle stimulating hormone results, etc.) as well as data on final diagnosis and management. Additional data on patients’ outcome were collected when available.

The study received approval from the Local Ethics Committee.

## RESULTS

We identified 10 cases of PHP type 1A with a mean age at diagnosis of 41.1 months (range from 4 to 156 months). There were 5 boys and 5 girls. Patients’ characteristics, clinical signs at presentation, and family medical history are summarized in Table 1. The family history was positive for AHO in mothers of 4 patients (cases 1, 5, 6, 7). Six mothers were short with a mean height of 145.8 cm (<-3.13 SDS) (cases 1, 2, 5, 6, 7, 10). Birth data were available for all 10 children, the mean gestational age being 38.5 weeks (range 37 to 40 weeks).

The study population’s mean BW was 2873±607.09 g, with a mean BL of 42.15±14.92 cm. Mean BW was lower in boys in comparison to girls [2838 g (-1.67 SDS) versus 2904 g (-1.0 SDS), respectively] (p=0.85). Girls were, however, shorter at birth, with a mean BL of 45.7 cm (-2.05 SDS) versus 48.25 cm (-0.90 SDS) in boys (p=0.55). Moreover, 4 out of 5 girls were small for gestational age for BL.

Our study population’s mean BMI was 21.7±3.94 kg/m2. At the time of diagnosis, seven children were overweight, with BMI above 85^th^ centile (16), their mean age was 14.6 months (range 5 to 32 months). Rapid weight gain calculated as an upward increase in body weight ≥0.67 SDS during infancy was noted in six children (cases 1, 2, 3, 5, 6, 8); in one case (patient 9), rapid gain in weight was recorded during early childhood. This gain in weight in early life was suggestive of early overweight and/or obesity in most children.

In children aged less than two years, the predominant clinical signs were obesity (70%) and subcutaneous ossifications (60%) diagnosed at a mean age of 17.3 months (range 5 months to 5 years), whereas in older children, brachydactily was present in 60% of cases (age at diagnosis 96.5 months-range 50 to 156 months). 

As shown in Table 2, erythrocyte Gsα activity was studied in six patients and the results were frankly suggestive of PHP in all these cases (activity <85%).

GNAS1 gene molecular testing was performed in 9 patients and the results revealed de novo mutations in three cases (c.585delGACT in exon 8) (case 2) and (c.344C>TP115L in exon 5) (cases 6&7). In the remaining children, mutations were maternally inherited. The location of different mutations identified in our patients is shown in Table 2.

Biochemically, four children presented with hypocalcemia (cases 2, 4, 5, and 9), their mean age at diagnosis was 69.3 months (range 8 to 108 months). Only one of these patients was aged <2 years (case 5), confirming that this abnormality has a progressive onset. Serum PTH levels were increased in 83% of our patients before the age of two years, but this early rise was not associated with hypocalcemia. Mean age at diagnosis of rise in PTH in our study population was 27.8 months (range 1 to 108 months). Hyperphosphatemia was diagnosed in 50% of cases. Subclinical hypothyroidism [high TSH with normal or low free thyroxine (fT4)] was present in 70% of the cases and was diagnosed at a mean age of 38 months (range 6 days to 168 months).

Seizures occurred in older children over age two years (cases 2, 4, and 9) at a mean age of 5.5 years.

GH status was tested only in one patient (case 6), in whom results were in favour of GH deficiency (peak serum GH <20 mU/L). None of our older patients was diagnosed with hypergodotropic hypogonadism, the younger patients were, unfortunately, not all tested.

## DISCUSSION

This study aimed at describing those early clinical features that should lead clinicians to consider PHP type 1A as a potential diagnosis in a child.

The commonly reported clinical features in children with PHP are lunar face (70%), short stature (80%), obesity (up to 90%), brachymetacarpy (70%), subcutaneous ossifications (42%), and a variable degree of mental retardation (64%) (17,18). All these features are rarely present together in a given patient in the early stage of the disorder which is often the reason why the diagnosis of PHP type 1A is made more difficult and often delayed.

Our results seem to indicate that the presenting clinical features characteristic of PHP type 1A are age-dependent. Taken together with those previously reported (8), it clearly appears that PHP type 1A has a bimodal clinical and biological presentation. Clinical features such as lunar face, short stature, subcutaneous ossifications, and obesity were most predominant signs in toddlers (age <2 years) and should raise suspicion. It is of note that in some patients, the search for subclinical ossifications may sometimes require X-ray investigation, as suggested by Elli et al (19,20).

Seizures as an inaugural manifestation reported by others (21) occurred in only three children whose mean age was 5.5 years (cases 2, 4, and 9) in our series. Despite being the primary biochemical abnormality in PHP type 1A, hypocalcemia with its various clinical expressions (muscle weakness, seizures, etc.) occurs secondary to the rise in PTH. An elevated PTH level, however, is rare in early infancy. Moreover, even in toddlers with raised serum PTH levels, hypocalcemia and seizures were found to be rare, with delayed clinical expression. The reasons for this delayed occurrence has recently been provided in a recent study by Turan et al (22) who were able to demonstrate, in knockout mice model, that manifestations of PTH resistance caused by maternal loss of Gαs develop after early postnatal life. On the other hand, the silencing of the paternal Gαs allele in proximal renal tubules is delayed in onset and gradual. This delay in imprinting of GNAS in proximal renal tubules leads to delay in PTH resistance and also to the clinical and biochemical expression of its associated manifestations such as hypocalcemia and seizures (22). We, additionally, speculate that vitamin D and/or calcium supplementation in toddlers, as practiced in some countries, could also play a role in delaying the occurrence of hypocalcemia in some patients.

Brachydactyly defined by the shortening of the metacarpals 3, 4, and 5, although typical of PHP, is a progressive sign (23) and is also a non-specific finding in the general population (1). When present, this sign was apparent in only 60% of our patients, all of whom were aged 5 years and above (mean 7.6 yrs at diagnosis), as also reported earlier by Fernandez-Rebollo et al (9). The fact that we did not calculate the metacarpophalangeal pattern profile in all our patients, as suggested by Poznanski et al (23), could have led to an underestimation of the true prevalence of this typical sign of PHP (23,24).

Obesity is a common finding in children with PHP type 1A. We evaluated the variation in body weight in our study population by comparing the gain in weight during infancy and early childhood and found that in most of our patients (7/10, 70%), that upward weight gain was greater than ≥0.67, a rise that is considered an early marker of obesity (17,25). Our findings corroborate previous reports (26,27) and indicate that in unexplained early obesity in a child, PHP type 1A should be considered. It is noteworthy that two of our patients (cases 1 and 3) were brought to medical attention with rapidly increasing body weight as the main complaint at the age of six months (BMI 21.43 kg/m2) and three months (BMI 22.6 kg/m^2^), respectively.

Long et al (25) have reported that obesity is more severe in patient when the mutant allele is inherited from the mother. When the mutant allele is paternal in origin, as it is in pseudopseudohypoparathyroidism, obesity is often not present or it is less severe.

As this study was retrospective, we were not able to perform additional tests to ascertain the precise origin of the mutant allele and hence analyze the possible correlation between the mutant allele and the degree of obesity.

With the pathognomonic signs of PHP becoming more apparent at different ages, our findings confirm that this disorder is a heterogeneous disease with variable clinical presentation.

Biochemically, the earliest features suggestive of PHP type IA in our population were subclinical hypothyroidism characterized by high TSH with normal or low fT4 present in 70% of cases, followed by increased PTH (83% of cases) and hyperphosphatemia (50% of cases). These findings were similar to those reported by others (9).

Moreover, two patients in this study (cases 8 and 9) had presented in the neonatal period with prolonged jaundice associated with increased TSH [26 mUI/L and 27 mUI/L, respectively (Normal range 0.3-5.0 mU/L)].

In spite of subclinical hypothyroidism being an important feature in the mode of presentation of PHP type 1A, none of the patients had been detected by the systematic post-natal congenital hypothyroidism screening. Whether lowering the TSH screening cut-off point would have detected affected children is difficult to ascertain in a retrospective study like this one. Langham et al (28) have recently reported that lowering TSH screening cut-off point to >6 mU/L as practiced at Great Ormond Street Hospital in London enabled their team to diagnose up to 36% of children with transient hypothyroidism who were subsequently treated. Further prospective studies addressing the ideal TSH screening cut-off point that would detect the largest number of children with thyroid pathology, including those with PHP is awaited.

As previously reported by Riepe et al (29), we overall found the association of early subclinical hypothyroidism with rapid gain in weight and more or less subcutaneous ossifications to be the most prominent clinical features. Our results suggest, therefore, that a child with subclinical hypothyroidism and a rapid increase in body weight should be investigated for PHP.

The diagnosis of this disorder is based on clinical and biochemical findings, as well as on the molecular study of GNAS gene. Molecular biology study results were available for nine of our patients. Known heterozygous mutations were found in six children (cases 1,3,4,5,8, and 10) in exons 1,5,7,9, and 12; in four patients for whom maternal DNA study had been performed, identical mutations to those found in their children were present (cases 1, 3, 5, 8). In three other children (cases 2, 6, and 7), mutations were de novo-c.delGACT in exon 8 (case 2) and c.344C>TP115L in exon 5 (cases 6 and 7, brother, sister, and their mother).

Lastly, we looked into the existence of a possible correlation between the genotype/phenotype and the age at onset of clinical landmarks of PHP type IA in patients with various mutations detected in our patients; we could not find any specificity and/or significant difference. No difference was found between our patients and those carrying similar mutations as published in the literature either (9).

In conclusion, PHP type 1A is a rare and complex condition, but it has some clinical features that should raise suspicion. Diagnosing this disorder can be tricky as characteristic clinical and biochemical parameters do not follow a similar chronological pattern in all patients. Based on our results, one should take into account two different periods each with its related signs, namely, round face, rapid weight gain, subclinical hypothyroidism, and subcutaneous calcifications in toddlers, and moderate mental retardation, brachydactyly, afebrile seizures (hypocalcemia), short stature, and TSH resistance in older children. Patients presenting with these associations should be screened for PHP type IA and close clinical follow-up organized thereafter.

## Acknowledgment

We are thankful to Dr Catherine Boniface (Saintes), Dr. Sophie Troller (La Rochelle), and Dr. Ariane Zelinski (Niort) for allowing us to analyze their medical records and include patients into this study.

## Ethics

Ethics Committee Approval: The study received approval from the Local Ethics Committee, Informed Consent: Retrospective study.

Peer-review: Externally peer-reviewed.

## Figures and Tables

**Table 1:**
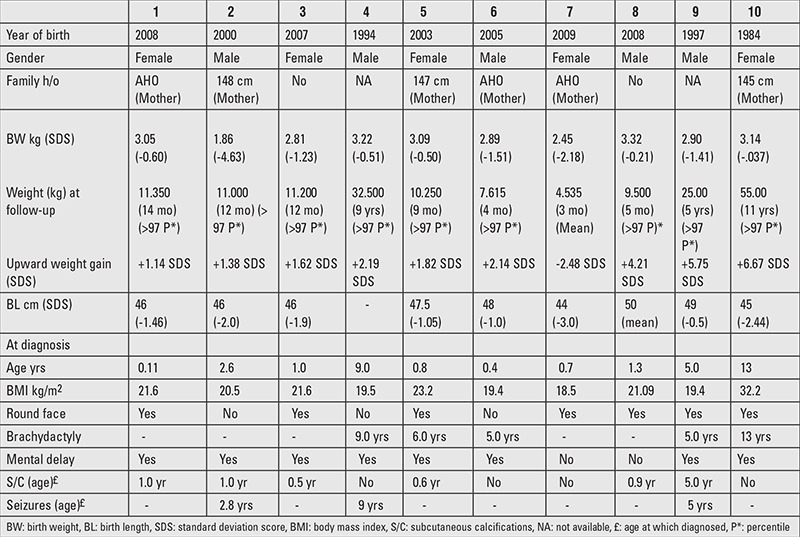
Patients’ characteristics, ages, signs at diagnosis, and family history

**Table 2:**
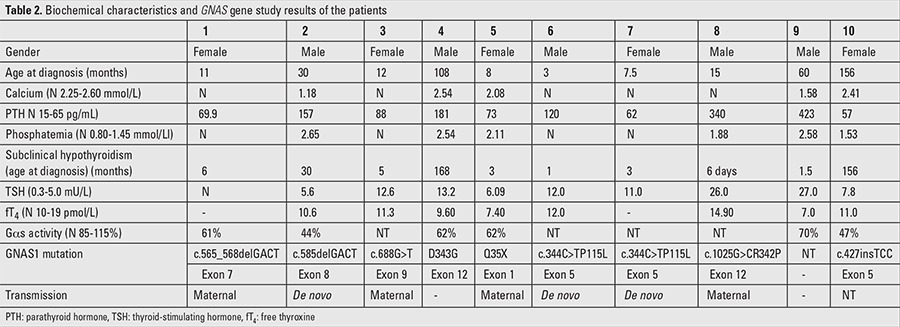
Biochemical characteristics and GNAS gene study results of the patients
